# Correlation analysis between CT values of cochlear bone labyrinth and auditory function and disease progression in patients with chronic suppurative otitis media

**DOI:** 10.3389/fmed.2026.1789884

**Published:** 2026-05-19

**Authors:** Shaoguang Ding, Xiaodong Jia, Cuncun Xie, Xiaoli Ding, Hongjian Liu

**Affiliations:** Department of Otorhinolaryngology-Head and Neck Surgery, Henan Provincial People’s Hospital, People’s Hospital of Zhengzhou University, People’s Hospital of Henan University, Zhengzhou, Henan, China

**Keywords:** auditory function, bone loss, chronic suppurative otitis media, cochlea, CT value

## Abstract

**Objective:**

To investigate the correlation between CT values of cochlear bone labyrinth (HU, as a radiological alternative indicator of bone mineral density) and auditory function and disease progression in patients with chronic suppurative otitis media.

**Methods:**

A retrospective collection of clinical data was conducted on 128 patients diagnosed with chronic suppurative otitis media who visited the Otolaryngology Department of our hospital from May 2023 to May 2025. All patients underwent high-resolution CT scans of the temporal bone. Parallel pure tone audiometry (including 0.5, 1, 2, and 4 kHz air and bone conduction thresholds) and speech recognition rate testing were performed. Pearson analysis was used to investigate the correlation. A multiple linear regression model was carried out to analyze independent factors.

**Results:**

The CT values of the affected ear in the basal transition area of the cochlear bone labyrinth were significantly lower than the healthy ear (*P* < 0.001). Patients with cholesteatoma type CSOM had lower ear basal to CT values compared to patients with simple CSOM (*P* < 0.001). There was a negative correlation between the basal to CT values of the affected ear and the bone conduction hearing thresholds at various frequencies, and the correlation was increased with increasing frequency (*P* < 0.001, 0.5 kHz: *r* = −0.355; 4 kHz: *r* = −0.521). The CT value was positively correlated with speech recognition rate (*r* = 0.427, *P* < 0.001), and significantly negatively correlated with disease duration (*r* = −0.430, *P* < 0.001). After adjusting for other influencing factors, disease duration (β = −11.284, *P* < 0.001), age (β = −7.693, *P* = 0.001), age of initial treatment (β = 5.827, *P* = 0.010), and type of otitis media (β = −48.326, *P* = 0.003) were independent factors associated with basal to CT values. In addition, basal to CT value (β = −0.018, *P* < 0.001), disease duration (β = 0.517, *P* < 0.001), age (β = 0.284, *P* = 0.004), and type of otitis media (β = 6.194, *P* = 0.002) were independent factors correlated with bone conduction hearing threshold (4 kHz).

**Conclusion:**

The course of illness, age, and presence of cholesteatoma are independent factors associated with CT values and hearing loss. Changes in the bony structure of the inner ear and early intervention may help protect auditory function.

## 1 Introduction

Chronic suppurative otitis media (CSOM) is a prevalent otological disorder characterized by persistent or recurrent inflammation of the middle ear cleft, involving the mucosa, periosteum, and underlying osseous structures. This chronic inflammatory state frequently results in tympanic membrane perforation, middle ear effusion, and progressive structural erosion (1). Absent timely clinical intervention, the inflammatory process may extend to the mastoid bone, potentially precipitating severe intracranial and extracranial sequelae (2). While existing literature has extensively explored microbiological profiles, inflammatory mediators, and surgical outcomes, the histopathological alterations within the inner ear—specifically the cochlear bony labyrinth—remain inadequately characterized (3, 4). The cochlear labyrinth is fundamental to auditory transduction; thus, subtle fluctuations in CT value (HU, which serves as a radiological substitute indicator for bone mineral density) may compromise the mechanical coupling of hair cells, thereby impairing hearing thresholds and frequency discrimination. Furthermore, localized CT value reduction may reflect an interplay between chronic inflammation and systemic bone metabolism (5).

Clinically, early stage CSOM typically manifests as conductive hearing loss. However, as the inflammatory process progresses toward the inner ear, hearing impairment often gradually manifests as sensorineural component, resulting in complex mixed hearing loss (6). Evidence suggests that elevated bone-conduction (BC) thresholds in CSOM patients signify cochlear dysfunction or impaired inner ear transmission, which often correlates with disease chronicity (7, 8). The speculated mechanisms include the direct ototoxicity of inflammatory cytokines on hair cells and alterations in the cochlear microenvironment mediated by increased round window membrane permeability. Additionally, chronic inflammation may disrupt the local bone metabolic milieu, altering the mechanical and acoustic properties of the cochlear labyrinth (9).

Despite these observations, clinical evidence remains insufficient regarding the association between regional cochlear CT value alterations and frequency-specific auditory deficits. Consequently, this study aims to quantify CT value across distinct cochlear regions in CSOM patients and evaluate its correlation with frequency-specific hearing thresholds, speech recognition scores, and disease duration. Through multivariate regression analysis, we seek to identify the independent factors related to CT value reduction and auditory impairment, thereby establishing a theoretical framework for targeted therapeutic interventions and hearing preservation in CSOM.

## 2 Materials and methods

### 2.1 Study population

**FIGURE 1:**
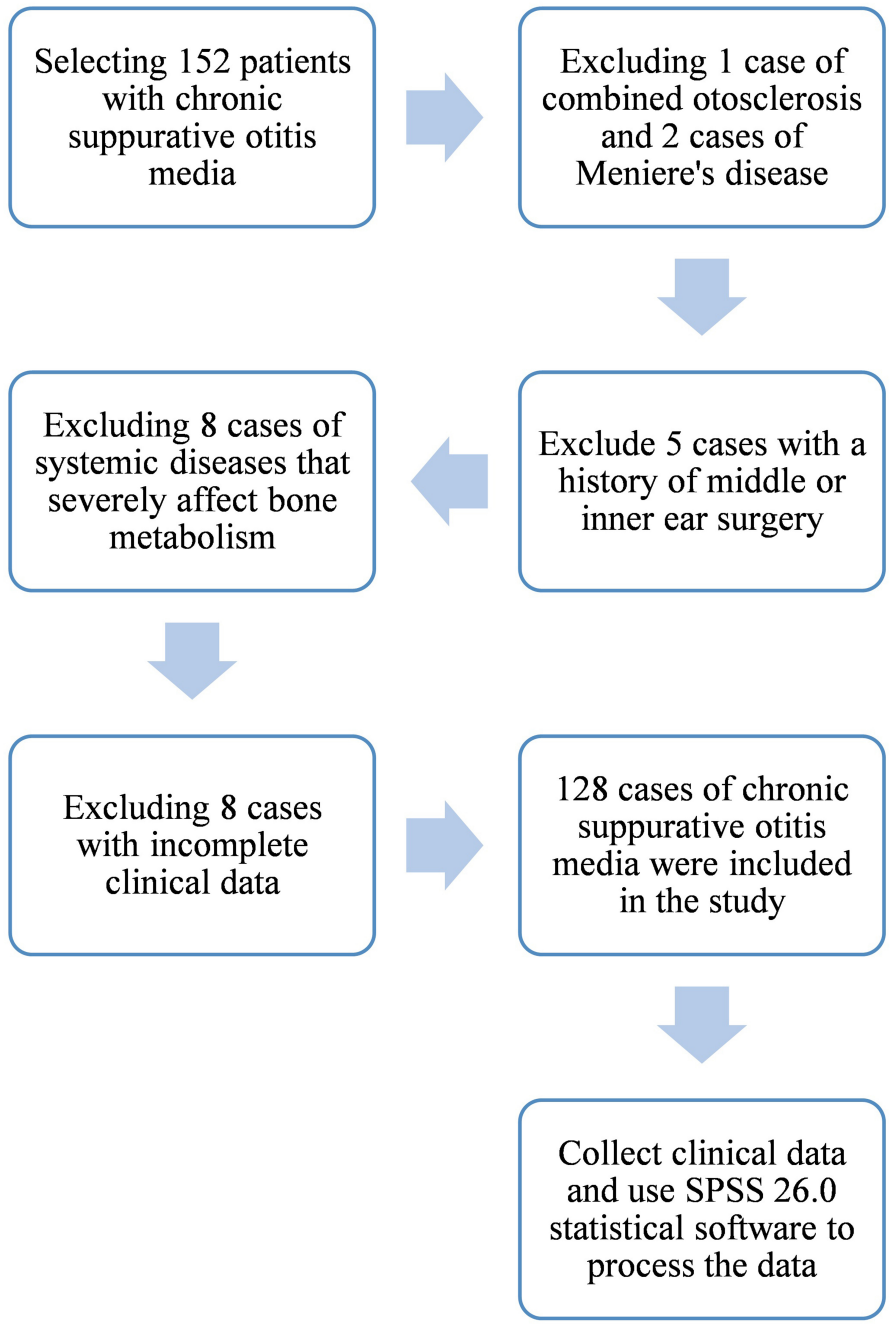
Flowchart illustrating the patient screening process.

This retrospective study analyzed clinical data from patients diagnosed with chronic suppurative otitis media (CSOM) at our institution between May 2023 and May 2025. From an initial pool of 152 screened candidates, 128 patients were enrolled following the application of strict eligibility criteria (Figure 1).

Inclusion Criteria: (1) Diagnosis of CSOM according to established guidelines (10), specifically: (a) persistent or intermittent otorrhea lasting > 3 months; (b) tympanic membrane perforation confirmed via otoscopy; and c) conductive or mixed hearing loss verified by pure-tone audiometry (PTA). (2) Age 18 years or above with full capacity for clinical cooperation. (3) Comprehensive clinical records, including detailed medical history, otoscopic imaging, and audiometric data.

Exclusion Criteria: (1) Concurrent otological pathologies affecting the inner ear or ossicular chain (e.g., otosclerosis, Meniere’s disease). (2) History of middle or inner ear surgery. (3) Prior head trauma involving temporal bone abnormalities. (4) Systemic metabolic bone disorders (e.g., hyperthyroidism, parathyroid dysfunction, renal insufficiency) or long-term corticosteroid therapy. (5) Severe systemic comorbidities with an expected survival of < 1 year. This study was approved by the Institutional Ethics Committee, and all procedures were conducted in accordance with the Declaration of Helsinki.

### 2.2 Methods

#### 2.2.1 Clinical data acquisition

Clinical data were systematically collected by trained specialists through a review of electronic medical records and standardized face-to-face interviews. Collected variables included demographic information (age, sex), disease duration (calculated from the initial onset of aural symptoms), the side of the affected ear, history of prior treatments, and the preliminary clinical classification upon admission (e.g., the presence of cholesteatoma).

#### 2.2.2 High-resolution CT (HRCT) and bone mineral density assessment

All patients underwent high-resolution computed tomography (HRCT) of the temporal bone using a SOMATOM Scope 16-slice spiral CT scanner (Siemens, Germany). Scanning parameters were standardized as follows: tube voltage 120 kV, tube current 250 mA, slice thickness 0.625 mm, and slice spacing 0.3 mm. The scan range extended from the petrous apex to the mastoid tip to ensure comprehensive imaging.

Image data were processed using a Syngo via workstation. Two radiologists, blinded to patient grouping, independently measured CT values (Hounsfield Units, HU) to serve as an indirect radiological indicator of bone mineral density. Measurements were taken using a standardized bone window (width: 4,000 HU; level: 700 HU). A circular region of interest (ROI) with an area of 2 mm^2^ was manually placed on each selected level at four specific sites: (1) the axial section of the cochlear basal turn; (2) the middle turn; (3) the apical turn; and (4) the anterior wall of the internal auditory canal. To minimize the partial volume effect to the greatest extent and ensure the accuracy of ROI placement, all measurements were conducted on thin-layer (0.625 mm) axial images, and combined with coronal and sagittal multi-planar reconstructions (MPR) to precisely locate the optimal display level for each turn of the cochlea. The placement of ROI strictly follows the following principles: the measurement is limited to the cortical bone part of the ear capsule, avoiding the areas of bone trabeculae and any possible sclerosis or granulation tissue; by adjusting the position and size of ROI, ensure that it is completely within the bony contour, maintaining a minimum safe distance of at least 1 mm from the surrounding air (such as the middle ear cavity, mastoid air cells) or liquid spaces (such as the vestibule, tympanic scala, and inner ear canal) to avoid interference from volume effects; for affected ears with cholesteatoma, granulation, or sclerotic lesions, select areas with relatively intact and clearly distinguishable bony structures for measurement, and avoid the bone regions adjacent to the lesions. To ensure the standardization and repeatability of ROI placement, two radiologists received unified training before the formal measurement and practiced using 10 pre-experiment cases until they reached consensus. Each part of each patient was measured three times and the average value was used for subsequent analysis. The intraclass correlation coefficient (ICC) of the measurement results of the two physicians was calculated. In this study, the ICC of all measurement sites was > 0.90, indicating good measurement consistency.

In this study, the contralateral ear was selected as the control group, aiming to reduce the interference from confounding factors such as genetic background, age, gender, and overall bone metabolism status among individuals through self-control. At the same time, it was ensured that the affected ear and the contralateral ear were under the same imaging acquisition conditions to control the systematic error. Moreover, chronic suppurative otitis media is mostly significantly affected on one side, and the contralateral ear can often be used as a relatively normal reference in clinical practice (11, 12). As a control ear, the definition of “normal” for the contralateral ear was to meet the following criteria simultaneously: (1) Clinical criteria: no tympanic membrane perforation, no active inflammation, no cholesteatoma or granulation tissue detected by otoscopy; (2) Audiological criteria: Pure tone audiometry showed that the bone conduction hearing threshold at each frequency (0.5, 1, 2, 4 kHz) was ≤ 25 dB HL, and the air-bone conduction difference was ≤ 10 dB; (3) Radiological criteria: HRCT of the temporal bone showed no inflammatory changes in the middle ear and mastoid, no cholesteatoma, no destruction or fixation of the ossicular chain, and no abnormalities in the inner ear structure. All 128 patients in this study met the above criteria for their contralateral ear.

The HU value reflects the degree of attenuation of X-rays by the tissue. It is influenced by scanner parameters (such as tube voltage and tube current), reconstruction algorithms, and the placement position of the region of interest (ROI), and is not a direct measurement of bone mineral density.

#### 2.2.3 Audiological assessment

Audiological evaluations were conducted in a soundproof booth compliant with national standards. Using an AD226 pure-tone audiometer (Daner Hearing), air-conduction (AC) and bone-conduction (BC) thresholds were determined at 0.5, 1, 2, and 4 kHz frequencies. Hearing levels were recorded in decibels (dB). Speech recognition thresholds were assessed using the audiometer’s integrated speech testing function. A pre-recorded list of Chinese disyllabic words was presented at an intensity of 50 dB SL, and the speech recognition rate (SRR) was calculated as the percentage of words correctly repeated by the patient. All equipment was calibrated prior to the commencement of the study.

### 2.3 Statistical analysis

Data processing and analysis were performed using SPSS version 26.0 software. Quantitative data were tested for normality; variables conforming to a normal distribution (e.g., age, CT value, hearing thresholds) are expressed as mean ± standard deviation (x ± s). Intergroup comparisons were analyzed using paired sample *t*-tests. For comparisons involving multiple groups (e.g., different disease duration cohorts), a one-way analysis of variance (ANOVA) was employed when homogeneity of variance was satisfied, followed by LSD-*t* tests for pairwise comparisons. In cases where variance was unequal, Welch’s ANOVA was utilized, with Dunnett’s T3 test for pairwise comparisons. Categorical data are presented as frequency and percentage [n (%)] and were compared using the Chi-square (χ^2^) test. Pearson correlation analysis was used to evaluate the strength and direction of associations between variables (r). Multivariate linear regression models were constructed to identify independent factors influencing CT value and high-frequency hearing loss (4 kHz BC threshold). A *P*-value of < 0.05 was considered statistically significant.

## 3 Results

### 3.1 Patient demographics and clinical characteristics

A total of 128 patients with chronic suppurative otitis media (CSOM) were included in the final analysis, comprising 68 males (53.13%) and 60 females (46.88%). The cohort’s age ranged from 18 to 76 years, with a mean age of (45.32 ± 15.67) years. The mean disease duration was (15.73 ± 10.45) years (range: 1–40 years). Patients were stratified into three groups based on disease duration: short-duration (< 10 years, *n* = 42), medium-duration (10–20 years, *n* = 51), and long-duration (> 20 years, *n* = 35).

The mean age at initial treatment was (32.15 ± 14.28) years. The left ear was the affected side in 71 cases (55.47%). Conductive hearing loss was the predominant type (*n* = 85, 66.41%), while mixed hearing loss was observed in 43 cases (33.59%). Regarding CSOM subtypes, 85 cases (66.41%) presented with simple otitis media, and 43 cases (33.59%) were associated with cholesteatoma. Comprehensive demographic data are summarized in Table 1.

**TABLE 1:** Demographic and clinical characteristics of 128 patients.

Characteristics	Numerical value	Percentage (%)
Gender		
Male	68	53.13%
Female	60	46.88%
Age (years)		
18–40	52	40.63%
41–60	48	37.50%
> 60	28	21.88%
Average age (years)	45.32 ± 15.67	
Age at onset (groups)	32.15 ± 14.28	
Disease duration (years)		
< 10	42	32.81%
10–20	51	39.84%
> 20	35	27.34%
Average disease duration (years)	15.73 ± 10.45	
Affected side		
Left ear	71	55.47%
Right ear	57	44.53%
Types of hearing loss		
Conductive hearing loss	85	66.41%
Mixed hearing loss	43	33.59%
Types of otitis media		
Simple type	85	66.41%
With cholesteatoma type	43	33.59%

### 3.2 Regional cochlear CT value analysis

Comparison of CT value across different regions of the cochlear bony labyrinth between affected and contralateral (unaffected) ears is presented in Table 2. CT value in the basal turn of the affected ear was significantly lower than that of the contralateral ear (*P* < 0.001). Conversely, no significant differences were observed in the middle turn, cochlear apex, or internal auditory canal (*P* > 0.05). The overall mean CT value of the affected ear was significantly lower than that of the contralateral ear (*P* < 0.05), suggesting that CSOM-induced bone density reduction is primarily localized to the basal turn.

**TABLE 2:** Comparison of CT value in different cochlear regions between affected and contralateral ears (Unit: Hounsfield Units, *n* = 128).

Anatomical region	Affected ear (HU)	Contralateral ear (HU)	*T-value*	*P-value*
Basal turn	1542.28 ± 235.67	1672.41 ± 198.31	4.780	<0.001
Middle turn	1620.59 ± 247.15	1663.70 ± 218.57	1.478	0.141
Apical turn	1767.30 ± 298.91	1735.67 ± 245.70	0.925	0.356
Internal auditory canal	1857.54 ± 301.24	1872.34 ± 283.53	0.405	0.686
Overall mean CT value	1681.77 ± 215.63	1740.31 ± 198.72	2.259	0.025

Inter-group comparisons based on disease duration (Table 3) revealed a progressive decline in basal turn CT value as disease duration increased (*P* < 0.001). No significant differences were found in the CT value of the middle turn, apex, or internal auditory canal across the duration groups (*P* > 0.05), indicating that prolonged inflammation specifically correlates with progressive demineralization of the cochlear basal turn.

**TABLE 3:** Comparison of cochlear CT value in affected ears stratified by disease duration (Unit: Hu).

Anatomical region	Short duration (< 10 years) (*n* = 42)	Medium duration (10–20 years) (*n* = 51)	Long duration (> 20 years) (*n* = 35)	*F*-value	*P*-value
Basal turn	1618.42 ± 205.35	1506.73 ± 197.18*	1410.16 ± 185.79*^#^	10.79	<0.001
Middle turn	1658.27 ± 239.42	1615.84 ± 251.36	1578.65 ± 248.91	1.00	0.370
Apical turn	1789.52 ± 287.43	1762.18 ± 305.67	1738.25 ± 301.24	0.28	0.753
Internal auditory canal	1872.69 ± 295.38	1853.42 ± 308.15	1842.18 ± 299.67	0.10	0.903
Overall mean CT value	1714.73 ± 208.40	1672.54 ± 219.38	1634.31 ± 217.82	1.34	0.265

*P < 0.05 compared with the short disease duration group. ^#^*P* < 0.05 compared with the medium disease duration group.

To further clarify the impact of different types of otitis media on the basal turn CT value, a subgroup analysis was conducted based on simple chronic suppurative otitis media (CSOM) and CSOM with cholesteatoma. The results (Table 4) showed that the basal turn CT value in the affected ears of CSOM patients with cholesteatoma were significantly lower than patients with simple CSOM (*P* < 0.001). This suggested that the presence of cholesteatoma was associated with more obvious changes in the basilar transverse bone.

**TABLE 4:** Comparison of basal transverse CT values (HU) of the injured ears in different types of otitis media.

Types of otitis media	Case	CT value (HU) of basal turn	*T*-value	*P*-value
Simple CSOM	85	1598.34 ± 210.27	4.447	< 0.001
CSOM with cholesteatoma	43	1426.51 ± 198.64		

### 3.3 Audiometric outcomes and auditory function

Audiometric indicators for affected and contralateral ears are detailed in Table 5. Both air-conduction (AC) and bone-conduction (BC) thresholds across all tested frequencies were significantly higher in the affected ear (*P* < 0.001), with the most pronounced elevation observed at the high-frequency range (4 kHz). Furthermore, speech recognition scores were significantly lower in the affected ear compared to the contralateral ear (*P* < 0.001).

**TABLE 5:** Comparison of auditory function between affected and contralateral ears.

Audiometric parameter	Affected ear	Contralateral ear	*T*-value	*P*-value
Air-conduction threshold (dB HL)				
0.5 kHz	45.32 ± 12.69	18.42 ± 6.30	21.481	< 0.001
1 kHz	48.76 ± 13.40	16.70 ± 5.87	24.794	< 0.001
2 kHz	52.34 ± 18.28	19.54 ± 8.43	18.435	< 0.001
4 kHz	55.60 ± 16.37	21.33 ± 7.14	21.710	< 0.001
Bone-conduction threshold (dB HL)				
0.5 kHz	22.49 ± 8.30	15.69 ± 8.21	6.590	< 0.001
1 kHz	25.64 ± 9.12	16.31 ± 7.40	8.988	< 0.001
2 kHz	28.30 ± 10.26	17.87 ± 8.69	8.777	< 0.001
4 kHz	31.56 ± 11.44	19.46 ± 6.37	10.455	< 0.001
Speech recognition rate (**%)**	78.47 ± 12.30	95.63 ± 4.50	14.823	< 0.001

Analysis by disease duration (Table 6) demonstrated that AC and BC thresholds at all frequencies significantly increased with disease progression, while speech recognition rates (SRR) concurrently declined (all *P* < 0.05). These results suggest that auditory impairment in CSOM patients exacerbates in tandem with disease chronicity.

**TABLE 6:** Comparison of auditory function in affected ears stratified by disease duration.

Audiometric parameter	Short duration (< 10 years) (*n* = 42)	Medium duration (10–20 years) (*n* = 51)	Long duration (> 20 years) (*n* = 35)	*F*-value	*P*-value
Air-conduction threshold (dB HL)					
0.5 kHz	41.58 ± 8.24	46.13 ± 7.85*	49.86 ± 6.42*^#^	11.42	<0.001
1 kHz	44.92 ± 6.37	49.18 ± 5.62*	53.27 ± 6.08*^#^	18.58	<0.001
2 kHz	48.65 ± 10.35	53.82 ± 9.41*	58.46 ± 7.23*^#^	10.93	<0.001
4 kHz	51.73 ± 9.28	55.94 ± 10.52*	60.85 ± 7.16*^#^	9.18	<0.001
Bone-conduction threshold (dB HL)					<0.001
0.5 kHz	19.87 ± 6.45	23.15 ± 7.32*	26.38 ± 5.91*^#^	9.11	<0.001
1 kHz	22.46 ± 7.23	26.58 ± 6.24*	30.15 ± 9.87*^#^	9.63	<0.001
2 kHz	25.18 ± 4.41	29.42 ± 7.35*	32.85 ± 5.92*^#^	15.20	<0.001
4 kHz	28.34 ± 6.27	32.67 ± 8.38*	36.28 ± 7.15*^#^	11.11	<0.001
Speech recognition rate (%)	82.56 ± 9.45	77.83 ± 11.72*	72.15 ± 10.86*^#^	8.89	<0.001

*P < 0.05 compared with the short disease duration group. ^#^*P* < 0.05 compared with the medium disease duration group.

### 3.4 Correlation of CT value with auditory function and disease duration

Pearson correlation analysis (Table 7) revealed that basal turn CT value in the affected ear was negatively correlated with BC thresholds across all frequencies (*P* < 0.001). This negative correlation strengthened at higher frequencies, reaching its peak at 4 kHz (*r* = −0.521) compared to 0.5 kHz (*r* = −0.355). Basal turn CT value also showed a significant negative correlation with AC thresholds (*P* < 0.001), although the association was weaker than that observed with CT value thresholds. Additionally, CT value was positively correlated with SRR (*r* = 0.427, *P* < 0.001) and negatively correlated with disease duration (*r* = −0.430, *P* < 0.001).

**TABLE 7:** Correlation analysis of CT value with auditory function and disease duration (*n* = 128).

Variable	Correlation coefficient (r)	*P*-value
Correlation CT value with bone-conduction thresholds		
0.5 kHz	−0.355	< 0.001
1 kHz	−0.412	< 0.001
2 kHz	−0.468	< 0.001
4 kHz	−0.521	< 0.001
Correlation CT value with air-conduction thresholds		
0.5 kHz	−0.287	0.002
1 kHz	−0.326	< 0.001
2 kHz	−0.367	< 0.001
4 kHz	−0.399	< 0.001
CT value with speech recognition rate (%)	0.427	< 0.001
CT value with disease duration (years)	−0.430	< 0.001

### 3.5 Multivariate linear regression analysis

To identify independent predictors of basal turn CT value and 4 kHz BC thresholds, variables showing a significant association in univariate analysis (*P* < 0.1) or those clinically relevant were entered into a multivariate linear regression model. Multicollinearity was addressed by excluding variables with a variance inflation factor (VIF) > 5 using backward elimination.

The multivariate model (Table 8) identified disease duration (β = −11.284, *P* < 0.001), age (β = −7.693, *P* = 0.001), age at initial treatment (β = 5.827, *P* = 0.010), and otitis media subtype (β = −48.326, *P* = 0.003) as independent factors influencing basal turn CT value.

**TABLE 8:** Multiple linear regression analysis of factors affecting CT value and hearing loss (*n* = 128).

Dependent variable	Influencing factors	Regression coefficient (β)	Standardized coefficient	95% CI	*T*-value	*P*-value
Basal turn CT value						
	Constant term	1823.714	−		14.892	< 0.001
	Disease duration (years)	−11.284	−0.392	−16.695∼-5.873	−4.127	< 0.001
	Age (years)	−7.693	−0.298	−12.181∼-3.205	−3.284	0.001
	Age at initial treatment (years)	5.827	0.231	1.424∼10.230	2.614	0.010
	CSOM type (cholesteatoma)	−48.326	−0.273	−79.504∼-17.148	−3.045	0.003
Bone conduction hearing threshold (4 kHz)						
	Constant term	16.738	−		5.824	< 0.001
	Baseline CT value (Hu)	−0.018	−0.427	−0.025∼-0.011	−4.892	< 0.001
	Disease duration (years)	0.517	0.384	0.276∼0.758	4.263	< 0.001
	Age (years)	0.284	0.226	0.093∼0.475	2.937	0.004
	CSOM type (cholesteatoma)	6.194	0.271	2.300∼10.088	3.128	0.002

**FIGURE 2:**
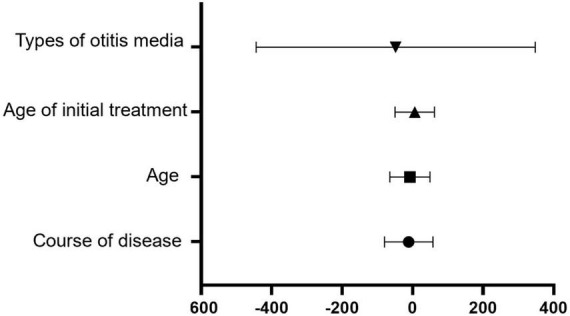
Forest plot illustrating the factors influencing basal turn CT value. Forest plot displayed the results of multiple regression analysis independently correlated with basal turn CT values. The horizontal axis represented the regression coefficient β and its 95% confidence interval, but the vertical axis represented the various influencing factors included in the regression model. A negative regression coefficient β indicated a negative correlation between the factor and the basal turn CT value, while a positive β illustrated a positive correlation. The error bar represented the 95% confidence interval. The error bar not cross the vertical line (β = 0) indicated that the association between this factor and the basal turn CT value was statistically significant (*P* < 0.05). This graph showed a negative correlation between disease duration, age, type of otitis media (with cholesteatoma), and basal turn CT value, but age at initial treatment was positively correlated with basal turn CT value.

**FIGURE 3:**
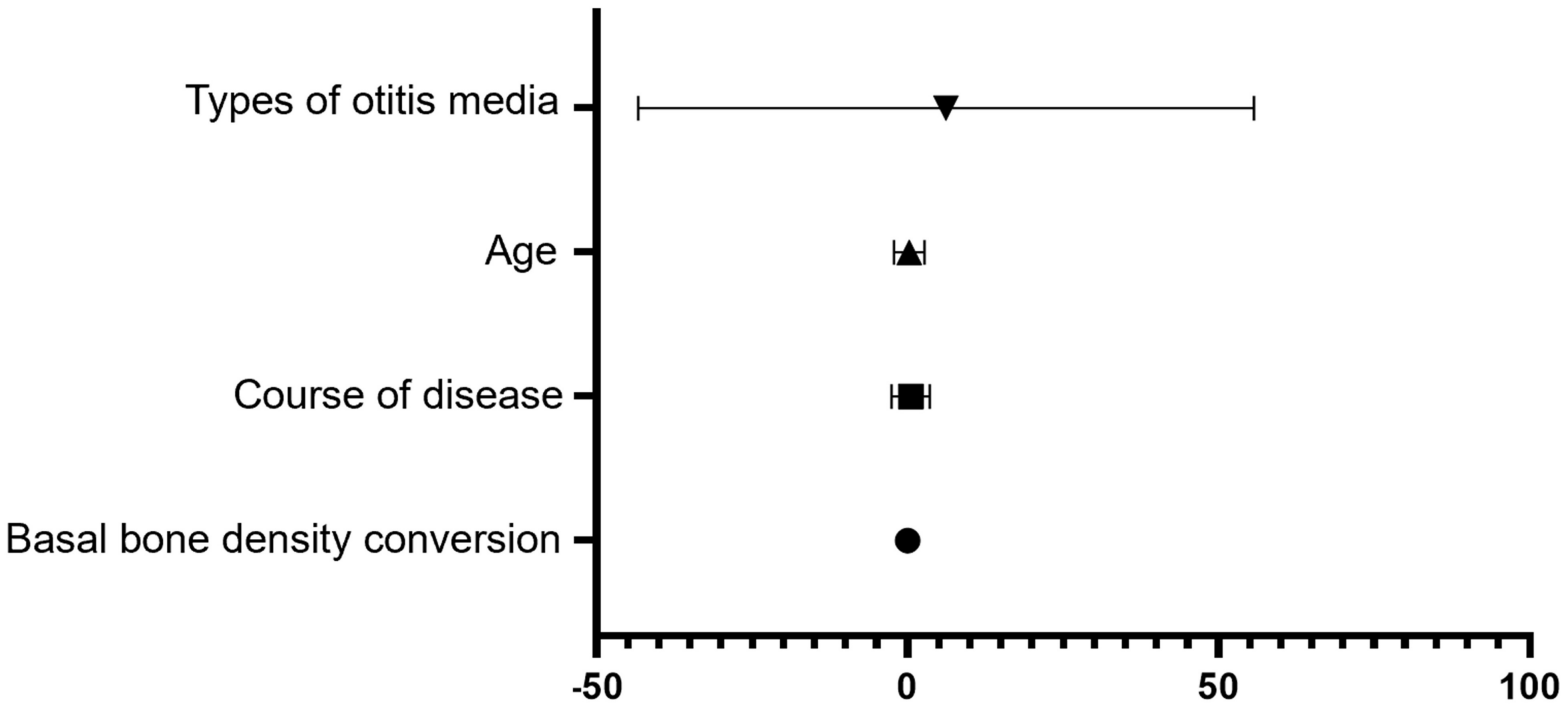
Forest plot depicting the factors affecting bone conduction hearing thresholds at 4 kHz. Forest plot displayed the results of multiple regression analysis independently correlated with bone conduction hearing threshold (4 kHz). The horizontal axis represented the regression coefficient β and its 95% confidence interval. The vertical axis indicated the various influencing factors included in the regression model. A positive regression coefficient β indicated a positive correlation between this factor and an increase in bone conduction hearing threshold. A negative coefficient β indicated a negative correlation. The error bar represented the 95% confidence interval. The error bar not cross the vertical line (β = 0) indicated that the association between this factor and bone conduction hearing threshold was statistically significant (*P* < 0.05). This graph showed that the course of disease, age, type of otitis media (with cholesteatoma), and elevated bone conduction hearing threshold were positively correlated, but basal turn CT value was negatively correlated with elevated bone conduction hearing threshold.

Furthermore, basal turn CT value (β = −0.018, *P* < 0.001), disease duration (β = 0.517, *P* < 0.001), age (β = 0.284, *P* = 0.004), and otitis media subtype (β = 6.194, *P* = 0.002) were identified as independent predictors of the 4 kHz BC hearing threshold (Figures 2, 3).

## 4 Discussion

The findings of this study indicate that patients with chronic suppurative otitis media (CSOM) exhibit a significant reduction in CT value within the cochlear bony labyrinth, particularly in the basal turn. This reduction is negatively correlated with the severity of hearing loss and the duration of the disease. Multivariate linear regression analysis identified disease duration, age, and the type of otitis media as independent factors associated with CT values and hearing loss. These findings offer a novel perspective on the mechanism of hearing loss in CSOM: beyond the traditional understanding of direct inflammatory toxicity and inner ear fluid disturbance, chronic inflammation may impair auditory function by disrupting the homeostasis of the bony microenvironment. This alteration affects the mechanical properties and acoustic transmission efficiency of the cochlear structure, which could provide a possible mechanism for the occurrence of sensorineural hearing loss.

Our results show that CT values in the cochlear basal turn of the affected ear was significantly lower than that of the healthy ear and exhibited a negative correlation with bone-conduction thresholds across all frequencies. This correlation was particularly robust in the high-frequency region (4 kHz), it was suggested that chronic suppurative otitis media may be associated with hearing impairment by affecting the integrity of the bony structure of the cochlea. The proposed mechanism might be that the release of inflammatory mediators and cytokines due to long-term chronic inflammation, which activates osteoclast activity, triggering bone resorption and remodeling of the otic capsule, thereby altering the mechanical vibration characteristics of the inner ear (13). Furthermore, inflammatory mediators may diffuse into the inner ear via the round window membrane or microscopic channels in the bony labyrinth, inducing a localized inflammatory response that indirectly affects bone metabolism (14). The selective reduction of the basilar membrane bone density has a clear anatomical basis. As the main anatomical channel between the middle ear and inner ear, the round window membrane connects to the cochlear base at an angle of approximately 42°. This anatomical adjacency relationship makes the basilar turn the primary gateway through which inflammatory mediators from the middle ear spread to the inner ear (15). Under inflammatory conditions, the permeability of the round window membrane increases, and bacterial products and inflammatory cytokines such as IL-1, IL-6, and TNF-α can enter the perilymph through this pathway, spread along the basilar turn and cause local damage (16). Animal experiments have confirmed that in the model of chronic suppurative otitis media, the loss of hair cells mainly occurs in the basal turn region, and is highly consistent with the distribution of macrophage infiltration, suggesting that local immune inflammatory responses play a key role in basal turn damage (17). From the perspective of auditory physiology, the basilar turn is responsible for the encoding of high-frequency sounds. Its hair cells are more sensitive to metabolic damage. A decrease in the density of the basilar turn’s bone labyrinth may alter the local mechanical properties and affect the efficiency of high-frequency sound waves in transmitting to the hair cells (18). The combined effect of the aforementioned anatomical adjacency relationships, the diffusion pathways of inflammatory mediators, and the characteristics of high-frequency auditory encoding explains why the changes in the bone density of the basal turn are most significantly associated with the increase in the high-frequency bone conduction hearing threshold.

The subgroup analysis of this study revealed that the CT values of the cochlear basal turn in patients with chronic suppurative otitis media accompanied by cholesteatoma were significantly lower than those in patients with simple CSOM (*P* < 0.001). This finding suggests that the presence of cholesteatoma may be associated with more significant changes in the cochlear bone structure. The bone destruction characteristics of cholesteatoma have been confirmed in clinical studies. Tang et al. (19) compared CSOM patients with and without cholesteatoma, finding that the inner ear sensorineural damage was more severe in the cholesteatoma group, and it was independently related to the disease course and the increase in bone conduction hearing threshold. They speculated that the mechanism might be related to the osteolytic inflammatory mediators released by the cholesteatoma. Serban et al. (20) further confirmed that the levels of IL-1α, IL-6, and IL-8 in the serum and local tissues of patients with cholesteatoma-type CSOM were significantly elevated. These cytokines can activate osteoclasts and promote bone resorption. However, Heo et al. (21) investigated the role of the RANKL/OPG signaling pathway in the bone destruction of CSOM, proposing that other cytokines might directly activate osteoclasts. This provided a new perspective for the discussion of the molecular pathways of the bone resorption mechanism in CSOM in this article. Compared with the above studies, this research is the first to directly quantify the impact of cholesteatoma on the CT values of the cochlear bone labyrinth (particularly the basal turn), and directly compared the differences in bone density between the simple type and cholesteatoma type CSOM in the same anatomical region (basal turn), providing more direct imaging evidence for the bone destruction characteristics of cholesteatoma involving the bony structures of the inner ear. From an anatomical perspective, cholesteatoma often affects the upper tympanic cavity and the tympanic sinus, being adjacent to the round window area. The mechanical compression and inflammatory exudation generated by it are more likely to spread to the basal turn of the cochlea through the round window membrane or the tiny channels of the bony labyrinth (22). The above evidence collectively supports that the significant reduction in the CT values of the cochlear-basilar axis in patients with cholesteatoma-type CSOM is not a coincidence, but rather a specific manifestation of their stronger potential for bone destruction in the bony structure of the inner ear. Therefore, in clinical assessment, for patients with CSOM accompanied by cholesteatoma, even if the symptoms of the middle ear are not yet very severe, it is necessary to be vigilant about the potential changes in the inner ear bony labyrinth. The measurement of the CT value of the cochlear basal turn on imaging may provide an auxiliary reference indicator for the risk of inner ear involvement in such patients.

Regarding auditory function, our results show that both air-conduction and bone-conduction thresholds were significantly elevated across all frequencies in the affected ear, with more severe damage observed in the high-frequency (4 kHz) bone-conduction thresholds. Further analysis of different disease duration groups found that as the disease course extended, thresholds at all frequencies increased significantly, while speech recognition rates synchronized in decline. These results collectively suggest that while the impact of CSOM on auditory function is dominated by conductive barriers in the early stage (elevated air-conduction thresholds), a sensorineural component gradually emerges and progressively worsens as inflammation spreads to the inner ear. It is speculated that the possible mechanism of this phenomenon is that inflammatory mediators and cytotoxins diffuse through the round window membrane or micro-channels of the bony labyrinth, directly invading the perilymphatic environment. This exerts toxic effects on the outer hair cells responsible for high-frequency perception in the basal turn and their associated nerve terminals, leading to functional impairment or apoptosis. Long-term inflammation may also damage the function of the stria vascularis and interfere with the ionic balance of the endolymph (such as potassium homeostasis), thereby impairing the endocochlear potential and weakening sensorineural function (23). Additionally, the decline in speech recognition rates indicates that the impact of CSOM on hearing extends beyond reduced sensitivity to involve higher-level auditory processing capabilities. This may be attributed to auditory deprivation caused by long-term hearing loss, which induces plastic changes in the central auditory pathways (24). Our results align with the report by Rasheed et al. (25), which noted that elevated bone-conduction thresholds in CSOM are directly proportional to the degree of inner ear damage. This suggests that in clinical evaluation, besides routine threshold testing, attention should be paid to assessing speech recognition abilities, which is significant for fully understanding the extent of auditory dysfunction and formulating reasonable rehabilitation plans.

Multivariate linear regression analysis results showed that disease duration, age, and otitis media type were independent factors correlated with the basal turn CT value. Disease duration represents the persistence of the condition; the longer the duration, the longer structural changes like inflammatory exudate, tympanic membrane perforation, or cholesteatoma may exert continuous mechanical stimulation on the basal turn, further promoting bone resorption. Consistent with the research conclusions of Philipose et al. (26), this study explored the determinants of cochlear dysfunction in CSOM mucosal-type diseases. It was found that the bone conduction hearing threshold of the affected ear was significantly higher than that of the healthy ear at all frequencies (0.5–4 kHz), and the high-frequency region was more severely affected. The disease duration and age were important influencing factors. Our study not only validates the negative correlation between duration and basal turn CT value but also further reveals the magnitude of this impact (β = −11.284) and clarifies its independent role in a multi-factor model. Moreover, by stratifying CT value changes across different cochlear regions, we found the basal turn to be more sensitive to the cumulative effects of chronic inflammation, expanding the understanding of the mechanism of disease duration in CT value changes and providing specific imaging evidence for early identification of high-risk groups. With increasing age, levels of estrogen or testosterone decline, leading to weakened bone formation and relatively enhanced bone resorption, causing a gradual decline in CT value (27). In cases of cholesteatoma, the enlarging mass produces mechanical compression while releasing cytokines like IL-6, TNF-α, PGE_2_, which activate osteoclasts; acidic environments and enzymes such as collagenase and matrix metalloproteinases (MMPs) further degrade the bone matrix, leading to rapid and localized bone destruction (28, 29). These findings suggest that the assessment of cochlear CT value may serve as an important biological marker for inner ear involvement in CSOM patients. Particularly for patients with a long disease course or recurrent attacks, regular temporal bone CT examinations and CT value assessment may help in the early detection of potential inner ear damage, providing a reference for the timing of clinical intervention.

Correlation analysis results in this study showed associations between CT value, multiple auditory function indicators, and disease duration. In addition to the strong negative correlation with bone-conduction thresholds mentioned earlier, the study also found that CT value was negatively correlated with air-conduction thresholds, though the correlation was weaker. This, together with the study by Kuru et al. (30), corroborates that CT value changes mainly affect inner ear sensorineural function, with a relatively limited impact on middle ear sound transmission structures. However, our study not only validated this association in the more focused cochlear labyrinth region but also revealed the quantitative law that the negative correlation between CT value and bone-conduction thresholds increases with frequency. Simultaneously, CT value was found to be positively correlated with speech recognition rates and significantly negatively correlated with disease duration. These results organically link microscopic structural changes in the inner ear (CT value), macroscopic auditory functional performance (speech recognition), and the temporal dimension of the disease (duration) into a complete chain of evidence. Previous studies mostly focused on the relationship between disease duration and pure-tone thresholds (31, 32), whereas this study further confirms that the degeneration of inner ear bony structures (reduced CT value) driven by long-term inflammation may be an important structural basis for the decline in speech understanding-a higher-order auditory function. This provides a new perspective for comprehensively assessing the damage to auditory function caused by chronic otitis media.

However, this study has certain limitations. Firstly, it is a cross-sectional design, which cannot establish a causal relationship between CT values and hearing loss, Secondly, this study used the HU value measured by HRCT as a substitute indicator for bone density, but the HU value may vary due to equipment, scanning parameters, and ROI selection. Future research should combine quantitative CT or microscopic imaging techniques to more accurately evaluate changes in bone density. Although we clearly set the inclusion criteria for the contralateral ear as the control ear in the method section (including clinical, audiological and radiological aspects), chronic suppurative otitis media may present with bilateral subclinical involvement. That is, although the contralateral ear does not have clear active manifestations of otitis media, there may still be mild inflammatory reactions or inner ear functional changes that are difficult to identify through routine examinations. Therefore, comparing the affected ear with the contralateral ear may underestimate the true extent of bone density changes, which is one of the methodological limitations of this retrospective study. Future research could use healthy volunteers as an external control group to more accurately assess disease-related changes. Furthermore, CT values in assessing bone density may involve certain errors. Future research could employ prospective designs, combine more precise bone density measurement techniques, and detect serum inflammatory factors to dynamically observe disease progression and deeply elucidate the relevant mechanisms.

## 5 Conclusion

In summary, patients with chronic suppurative otitis media exhibit significantly reduced CT value in the cochlear bony labyrinth, which is closely related to prolonged disease duration and the severity of bone-conduction hearing loss. Disease duration, age, and the presence of cholesteatoma are independent factors related to CT value and hearing loss. This suggests that in clinical management, in addition to controlling infection, attention should be paid to changes in the bony structure of the inner ear, and early intervention may help protect auditory function.

## Data Availability

The raw data supporting the conclusions of this article will be made available by the authors, without undue reservation.
